# Development of Duchenne Video Assessment scorecards to evaluate ease of movement among those with Duchenne muscular dystrophy

**DOI:** 10.1371/journal.pone.0266845

**Published:** 2022-04-13

**Authors:** Marielle G. Contesse, Linda P. Lowes, Michelle K. White, Laura Dalle Pazze, Christine McSherry, Lindsay N. Alfano, Megan Iammarino, Natalie Reash, Kelly Bonarrigo, Michael Kiefer, Katie Laubscher, Melissa McIntyre, Shelley Mockler, Leslie Nelson, Leslie Vogel, Mindy G. Leffler

**Affiliations:** 1 Casimir, Kingston, Massachusetts, United States of America; 2 Nationwide Children’s Hospital, Columbus, Ohio, United States of America; 3 Quality Metric, Johnston, Rhode Island, United States of America; 4 Charley’s Fund, New York, New York, United States of America; 5 Cincinnati Children’s Hospital Medical Center, Cincinnati, Ohio, United States of America; 6 MGH Institute of Health Professions, Boston, Massachusetts, United States of America; 7 University of Iowa Hospitals & Clinics, Iowa City, Iowa, United States of America; 8 University of Utah, Salt Lake City, Utah, United States of America; 9 University of Texas Southwestern Medical Center, Dallas, Texas, United States of America; 10 Seattle Children’s Hospital, Seattle, Washington, United States of America; Kaohsuing Medical University Hospital, TAIWAN

## Abstract

**Background:**

Patients with Duchenne muscular dystrophy (DMD) adopt compensatory movement patterns as muscles weaken. The Duchenne Video Assessment (DVA) measures patient ease of movement through identification of compensatory movement patterns. The DVA directs caregivers to video record patients performing specific movement tasks at home using a secure mobile application, and DVA-certified physical therapists (PTs) score the videos using scorecards with prespecified compensatory movement criteria. The goal of this study was to develop and refine the DVA scorecards.

**Methods:**

To develop the initial scorecards, 4 PTs collaboratively created compensatory movement lists for each task, and researchers structured the lists into scorecards. A 2-round modified Delphi process was used to gather expert opinion on the understandability, comprehensiveness, and clinical meaningfulness of the compensatory movements on the scorecards. Eight PTs who had evaluated ≥50 patients with DMD and participated in ≥10 DMD clinical trials were recruited for the panel. In Round 1, panelists evaluated compensatory movement criteria understandability via questionnaire and tested the scorecards. In Round 2, panelists participated in an in-person meeting to discuss areas of disagreement from Round 1 and reach consensus (≥75% agreement) on all revisions to the scorecards.

**Results:**

During the Round 1 revisions to the scorecards, there were 67 changes (44%) to the wording of 153 original compensatory movement criteria and 3 criteria were removed. During the Round 2 revisions to the scorecards, there were 47 changes (31%) to the wording of 150 compensatory movement criteria, 20 criteria were added, and 30 criteria were removed. The panel reached 100% agreement on all changes made to scorecards during Round 2.

**Conclusion:**

PTs with extensive experience evaluating patients with DMD confirmed that the compensatory movement criteria included in the DVA scorecards were understandable, comprehensive, and clinically meaningful.

## Introduction

Duchenne muscular dystrophy (DMD) is a rare, genetic disease characterized by progressive muscle degeneration and weakness that affects approximately 15.9 to 19.5 of every 100,000 live male births [1]. Genetic mutations in the dystrophin gene result in degeneration of muscle fibers involving inflammation and fibrosis, accompanied by reduced muscle regeneration. Progressive muscle weakness begins in early childhood and eventually leads to the loss of functional movement [2]. Death is often due to heart or respiratory failure [3, 4]. While there are a number of potential therapeutics being tested, there is still no cure for DMD [5–7]. Sensitive outcome measures that can be used in clinical trials for the development of potential therapeutics are critical.

People with DMD compensate for muscle weakness by changing their movement patterns [8–10]. Increased muscle weakness leads to a decline in ease of movement [11–13]. Many clinician-rated functional assessments used in DMD clinical trials are limited to differentiating between an inability to perform a test item, able to complete with compensations, or able to complete without compensations [14, 15]. They do not delineate between different severity levels of compensated movement for each test item. Measuring changes in the number or severity of compensations required to complete a movement task may allow clinical trials to detect incremental yet still clinically meaningful functional changes in a shorter duration of time. Clinicians, caregivers, and individuals with DMD consider needing assistance, using alternative techniques, and using more effort to complete a movement task as meaningful worsening in physical function [16]. When measuring such small increments of change, it will be important to ensure that each movement compensation acquired or lost represents a clinically meaningful amount of change in function.

**Fig 1:**
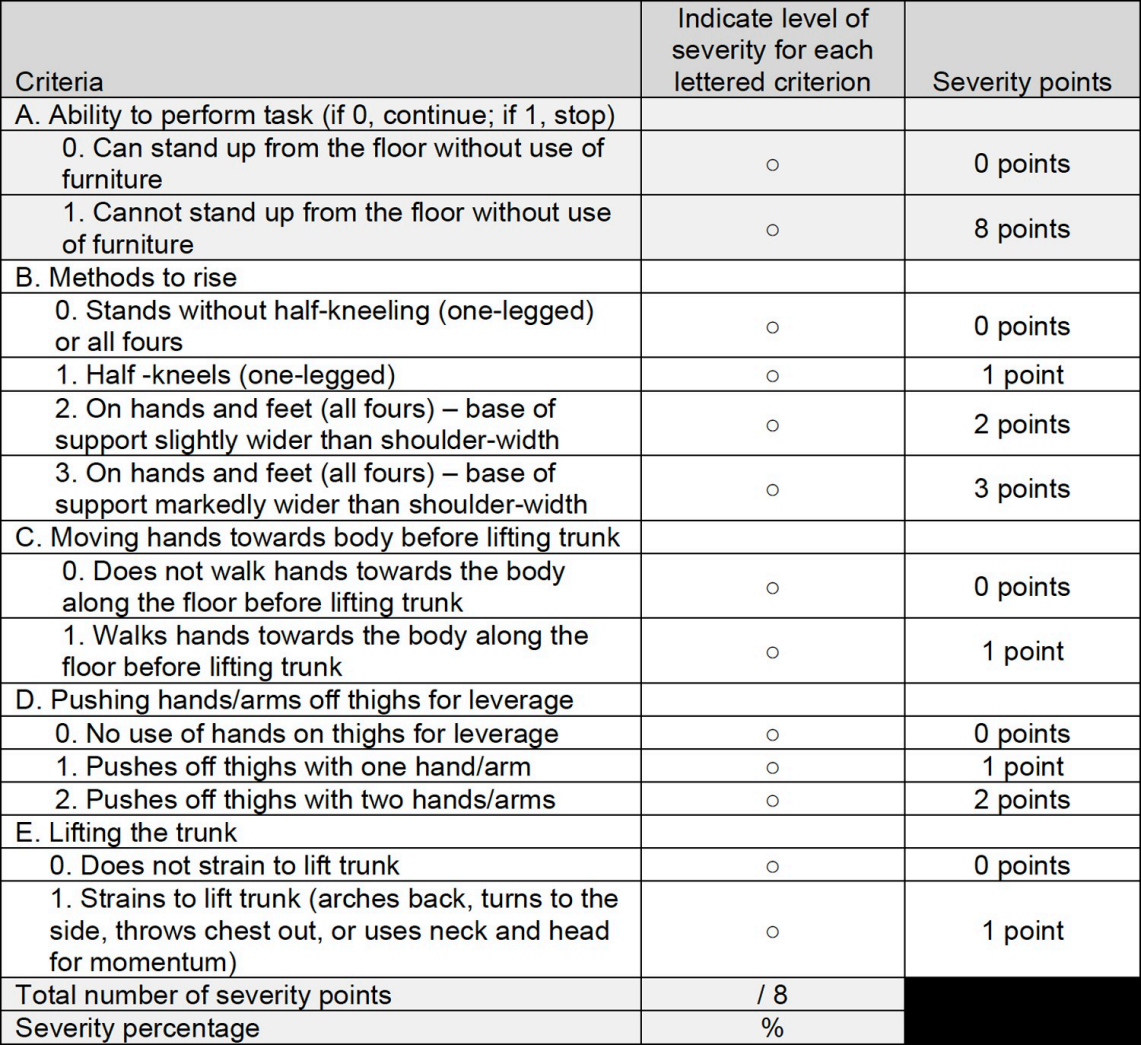
Duchenne Video Assessment scorecard for Stand Up from Sitting on the Floor. Within each compensatory movement criterion, raters select the sub-criterion that indicates the highest level of severity demonstrated in the video. For example, a compensatory criterion when standing up from sitting on the floor is: (D) “Pushing hands/arms off thighs for leverage”. The sub-criteria are: (0) no use of hands on thighs for leverage, (1) pushes off thighs with one hand/arm, and (2) pushes off thighs with two hands/arms. Task severity points are summed for an overall severity score. Not all tasks have the same number of possible points, so a severity percentage is calculated by dividing the severity score by the maximum severity score. Higher severity percentage indicates more compensated movement; the inability to perform a task is assigned the maximum severity score.

The Duchenne Video Assessment (DVA) is a novel, home-based clinical outcome assessment that measures ease of movement through identification of compensatory movement patterns. Rather than instructing best performance (e.g. “get up from the floor using as little support as possible and as fast as you can” [14] or “walk as far as possible for 6 minutes” [17]) in a clinic setting, the DVA evaluates typical performance in the home environment. The DVA directs caregivers to video record patients doing specific movement tasks at home using a secure mobile application. Caregivers are provided with a training manual and videos to standardize the set-up, lighting, clothing, surfaces, time of day, and instructions during video recording of movement tasks. The DVA movement tasks were selected through qualitative interviews with caregivers and clinicians to be relevant to patients with DMD in each disease stage and able to detect changes in function [18, 19]. The movement tasks are either foundational tasks for daily life (e.g. walking, climbing stairs) or activities of daily living (e.g. putting a t-shirt on, eating). Cognitive interviews with caregivers confirmed that the training materials and mobile application are easy to understand and use [18]. When the DVA is used in a research study, study staff monitor data collection, and caregivers are asked to re-record any videos that do not meet quality standards. The DVA videos are scored by DVA-certified physical therapists using scorecards (example provided in **Fig 1**) with prespecified compensatory movement criteria. The goal of this study was to develop and refine, using a modified Delphi approach, the DVA scorecards.

## Methods

### Source material

In a longitudinal study (Duchenne Video Project) of male participants with and without DMD, caregivers collected video data of participants performing DVA movement tasks for outcome measure development and testing. Detailed information about the Duchenne Video Project study population and methods is provided in another manuscript [20].

### Phase 1: Initial compensatory movement identification

In the first phase of scorecard development, four physical therapists from the Lowes Lab at Nationwide Children’s Hospital with extensive experience evaluating people with DMD contributed to the initial identification of compensatory movements for 15 movement tasks. They watched videos of approximately 25 Duchenne Video Project participants of varying abilities performing each movement task and independently created lists of the compensatory movements they observed for each task. They proceeded to discuss the compensations they observed and collaboratively developed preliminary lists of compensatory movements for each task.

### Phase 2: Scorecard formation

In the second phase of scorecard development, researchers who specialize in outcome measure development (M.C. and M.L.) structured the preliminary lists of compensatory movements into scorecards. Using the videos from the Duchenne Video Project, they observed the movement patterns of those without DMD and those of varying stages of DMD for each task and identified relationships between compensations and spectrums of severity. For example, using two hands for assistance when climbing the stairs is more severe than using one hand.

### Phase 3: Modified Delphi process

#### Study design and panel selection

**Fig 2:**
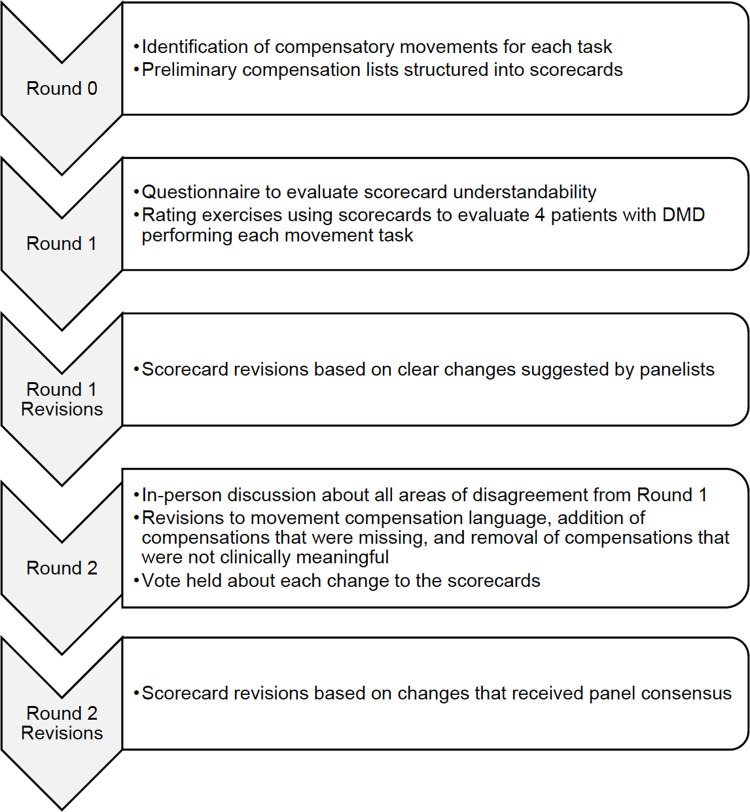
Modified Delphi process flow.

In the third phase of scorecard development, a 2-round modified Delphi process [21, 22] (**Fig 2**) was used to gather expert opinion on the scorecards from physical therapists who have evaluated a large volume of patients with DMD. The Delphi method [23] has often been used in health research to determine expert consensus for clinical problems and traditionally consists of iterative rounds of questionnaires with no direct interaction between panelists. The classic Delphi method has been criticized for not benefiting from the exchange of information between panelists that could identify reasons for disagreement [24]. This study used a modified Delphi process. The first round of the modified Delphi panel elicited qualitative feedback from panelists, and the identification of common themes allowed for fewer Delphi panel rounds than the classic method [25]. A second in-person round allowed panelists to explain disagreement and collaboratively revise the scorecards. The modified Delphi process benefited from both independent panelist evaluation of the scorecards in the first round and collaborative revision of the scorecards in the second round.

Eight panelists were recruited, consistent with the recommended modified Delphi method sample size [21], to be large enough to allow for diversity of experiences evaluating patients with DMD and small enough to allow all panelists to participate meaningfully in the group discussion. Physical therapists who had evaluated ≥50 people with DMD in clinic and participated in ≥10 DMD clinical trials were recruited from a list of United States physical therapists provided by a key opinion leader (L.L.). Only one of the physical therapists from Phase 1 participated in Phase 3. All participating physical therapists signed written agreements to provide consultant services for the purposes of the Delphi panel, and they were provided with an honorarium for their time.

Of the eight physical therapist panelists, three (38%) practiced physical therapy for ≥20 years, six (75%) provided physical therapy to ≥200 patients with DMD, and three (38%) participated in ≥15 DMD clinical trials (**Table 1**). All eight panelists participated in both Round 1 and Round 2 of the Delphi panel.

**Table 1:** Characteristics of physical therapists in panel.

Characteristic	Panelists
	N = 8
	n (%)
Gender	
Female	7 (87.5)
Male	1 (12.5)
Geographic Region	
U.S. Northeast	1 (12.5)
U.S. South	1 (12.5)
U.S. Midwest	4 (50.0)
U.S. West	2 (25.0)
Years of Physical Therapy Practice	
Fewer than 10	2 (25.0)
10–19	3 (37.5)
20 or more	3 (37.5)
DMD Patients Therapist has Evaluated	
Fewer than 200	2 (25.0)
200–399	3 (37.5)
400 or more	3 (37.5)
Number of DMD Clinical Trials	
10–14	5 (62.5)
15–19	2 (25.0)
20 or more	1 (12.5)

#### Modified Delphi process: Round 1

The intention in Round 1 was to revise language in the scorecards to improve understandability to physical therapists. For this round, panelists received the following via email: a DVA overview, preliminary questionnaire, and rating exercises. The DVA overview described how caregivers record videos, how physical therapists score videos, and how the preliminary scorecards were developed. The preliminary questionnaire included questions about physical therapy experience as well as an evaluation of the clarity of each DVA scorecard. For each scorecard, panelists indicated whether each compensatory movement criterion was easy to understand, understandable but needed modifications, or not understandable. If a compensatory movement criterion was not understandable or needed modification, the panelists provided a suggested change to improve clarity, which was included in the qualitative analysis.

For the rating exercises, the panelists watched videos of a different set of 4 participants from the Duchenne Video Project performing each movement task. Based on their clinical impressions, panelists ranked the 4 participants from strongest to weakest for the movement task. Next, panelists used the scorecard to evaluate each of the 4 participants. They were then asked whether the scorecard severity percentages reflected where the participants fell on the spectrum of strongest to weakest and to explain how the scorecards did or did not match up with their clinical impression of participant disease severity. Additionally, panelists were asked to report any problems they experienced while scoring the videos with the scorecards. The responses were included in the qualitative analysis.

The primary analyst (M.C.) compiled all qualitative suggestions for each criterion and coded suggestions as:

- Apply suggestion during Round 1: used when clear changes to movement compensation wording were suggested.
- Discuss suggestion during Round 2: used when unclear changes to movement compensation wording were suggested, a question was posed by the panelist about the movement compensation, or a suggestion conflicted with the suggestion of another panelist.
- Add detail to rater training materials to address suggestion: used when a panelist indicated that more background information was needed to understand a movement compensation.

The secondary analyst (M.L) reviewed all coded qualitative suggestions, and any coding disagreement between the two analysts was discussed and resolved.

The specific compensatory movement criteria selected for each participant’s video during the rating exercises were entered into an Excel spreadsheet, and all scoring discrepancies between panelists (e.g. one panelist selected sub-criterion B2 and seven panelists selected B1 for a participant’s video) were coded as “discrepant” by the primary analyst. The secondary analyst reviewed and confirmed the spreadsheet with criteria coded as “discrepant”. All participant videos with panelist scoring discrepancies were added to the Round 2 discussion slide deck for panel review and revisions to improve scoring reliability.

All qualitative suggestions coded as “apply suggestion during Round 1” were used to revise the scorecards prior to Round 2. A slide deck with each discussion item was created for Round 2, and a discussion worksheet was created to track the panel votes for each suggested modification to the scorecards.

#### Modified Delphi process: Round 2

The goals of the Round 2 meeting were to discuss all areas of disagreement from Round 1 and reach consensus (≥75% agreement) on all revisions to the scorecards. The primary and secondary analysts facilitated an 8-hour in-person meeting with the entire panel in Orlando, Florida on April 14, 2019. Ground rules were set that encouraged panelists to share their views openly during the discussion.

Panelists received copies of the original (Version 1) and revised (Version 2) scorecards at the beginning of the meeting. The facilitators presented the panel with fully anonymized feedback that required discussion from Round 1. The discussion during Round 2 focused on revising compensatory movement description language, adding compensations that were missing, and removing compensations that were not clinically meaningful. A vote was held about each decision during the in-person discussion, and the number of panelists who agreed with the change and the number of panelists who voted were recorded for each decision. Panelists were asked to keep in mind that the acquisition or loss of each compensatory movement on the scorecard should represent a clinically meaningful degree of change in function. At the end of the discussion for each scorecard, the panelists were asked if the scorecard was missing any compensatory movement criteria. The primary and secondary analysts revised the scorecards based on the changes made during Round 2 that received panel consensus.

## Results

### Modified Delphi process: Round 1

Based on panelist questionnaire responses, there were 67 changes (44%) to the wording of 153 compensatory movement sub-criteria in Version 1 (**Table 2**). As an example of the types of changes that were made to the scorecards, the specific revisions made to the Stand Up from Sitting on Floor scorecard are provided in **Table 3**. For that scorecard, the majority of panelists found all Version 1 sub-criteria except criterion A1 (“kneeling (one-legged)”) easy to understand, and A1 was revised based on the suggested wording of 4 (50%) of the panelists to become “half-kneeling (one-legged)”. All suggested wording changes for that scorecard were incorporated into the Round 1 revision, even if only a single panelist suggested a wording change.

**Table 2:** Revisions to scorecard compensatory movement criteria after each Delphi panel round.

	Original	Round 1 Revisions to Compensatory Criteria				Round 2 Revisions to Compensatory Criteria			
Scorecards	Total Version 1 Criteria	Word Changes	Added Criteria	Deleted Criteria	Total Version 2 Criteria	Word Changes	Added Criteria	Deleted Criteria	Total Version 3 Criteria
Climb 5 Stairs	**9**	7	0	0	**9**	1	2	2	**9**
Run	**8**	3	0	0	**8**	8	6	3	**11**
Walk	**13**	9	0	0	**13**	4	4	7	**10**
Jump	**17**	13	0	3	**14**	4	2	5	**11**
Sit Up	**11**	4	0	0	**11**	2	1	1	**11**
Stand Up from Sitting on Floor	**8**	5	0	0	**8**	2	0	0	**8**
Stand Up from Supine	**9**	5	0	0	**9**	2	0	0	**9**
Stand Up from Sitting on Couch	**7**	3	0	0	**7**	3	0	0	**7**
Raise Hands Above Head	**9**	3	0	0	**9**	6	2	1	**10**
Roll Over in Bed	**10**	0	0	0	**10**	3	2	2	**10**
Shift Weight in Bed	**10**	4	0	0	**10**	0	0	5	**5**
Take T-Shirt Off and Put T-Shirt On	**15**	1	0	0	**15**	7	1	1	**15**
Eat 10 Bites	**10**	4	0	0	**10**	4	0	0	**10**
Arms Off and On Armrests	**7**	4	0	0	**7**	1	0	3	**4**
Reach Across Table to Grab a Cell Phone	**10**	2	0	0	**10**	0	0	0	**10**
**TOTAL**	**153**	**67**	**0**	**3**	**150**	**47**	**20**	**30**	**140**

**Table 3:** Stand Up from Sitting on Floor scorecard revision tracking matrix.

Version 1	Round 1 Questionnaire		Round 1 Revisions	Version 2	Round 2 Revisions	Version 3
	Panelists					
	N = 8					
	n (%)					
	Criterion reported as “easy to understand”	Suggested specific wording change				
A. Starting position		1 (12.5)	Changed "starting position" to "methods to rise"	A. Methods to rise	Moved E to become A	A. Ability to perform task
1. Kneeling (one-legged)	3 (37.5)	4 (50.0)	Changed "kneeling" to "half-kneeling"	1. Half -kneeling (one-legged)	Added level 0 for all criteria	0. Can stand up from the floor without use of furniture
2. All fours (Gowers)–base of support slightly wider than shoulder width	7 (87.5)	1 (12.5)	Added "hands and knees" and removed "Gowers"	2. Hands and knees (all fours)–base of support slightly wider than shoulder width	Moved E to A1	1. Cannot stand up from the floor without use of furniture
3. All fours (Gowers)–base of support markedly wider than shoulder width	7 (87.5)	1 (12.5)	Added "hands and knees" and removed "Gowers"	3. Hands and knees (all fours)–base of support markedly wider than shoulder width	No changes (formerly A)	B. Methods to rise
B. Decrease distance between hands and feet before raising torso (e.g.: walking hands along floor)	6 (75.0)	1 (12.5)	Removed "decrease distance between hands and feet before raising torso"	B. Walking hands along floor	Added level 0 for all criteria	0. No half-kneeling (one-legged) or all fours
C. Use of hands for leverage		0 (0.0)	No changes	C. Use of hands for leverage	No changes (formerly A1)	1. Half -kneeling (one-legged)
1. Pushing off thighs (one hand)	6 (75.0)	0 (0.0)	No changes	1. Pushing off thighs (one hand)	No changes (formerly A2)	2. Hands and knees (all fours)–base of support slightly wider than shoulder width
2. Pushing off thighs (two hands)	6 (75.0)	0 (0.0)	No changes	2. Pushing off thighs (two hands)	No changes (formerly A3)	3. Hands and knees (all fours)–base of support markedly wider than shoulder width
D. Using alternate strategies to lift torso (Arch back, turning to the side, throwing chest out, using neck and head for momentum)	7 (87.5)	0 (0.0)	No changes	D. Using alternate strategies to lift torso (Arch back, turning to the side, throwing chest out, using neck and head for momentum)	No changes (formerly B)	C. Moving hands towards body before lifting torso
E. Inability to perform task	7 (87.5)	1 (12.5)	Added "without use of furniture"	E. Inability to perform task without use of furniture	Added level 0 for all criteria	0. No walking hands towards the body along the floor before lifting torso
					Added "towards the body" and "before lifting torso"	1. Walking hands towards the body along the floor before lifting torso
					No changes (formerly C)	D. Use of hands for leverage
					Added level 0 for all criteria	0. No use of hands for leverage
					No changes (formerly C1)	1. Pushing off thighs (one hand)
					No changes (formerly C2)	2. Pushing off thighs (two hands)
					No changes (formerly D)	E. Lifting the torso
					Added level 0 for all criteria	0. No straining to lift torso
					Changed "using alternate strategies" to "straining"	1. Straining to lift torso (Arch back, turning to the side, throwing chest out, using neck and head for momentum)

Three compensatory movement sub-criteria from the Jump Forward scorecard were collapsed into related sub-criteria during Round 1:

- Criterion A: The knee flexion in the squat position before the feet leave the ground was broken into two severity levels in Version 1: (1) limited knee bend and (2) no knee bend. Those two levels were collapsed into one criterion in Version 2: “Limited or no knee flexion”.
- Criterion G: The knee flexion in the landing squat was broken into two severity levels in Version 1: (1) limited knee bend and (2) no knee bend. Those two levels were collapsed into one criterion in Version 2: “limited or no knee flexion on landing squat”.
- Criterion H: The bend at the waist after landing was broken into two severity levels in Version 1: (1) slight bend at the waist and (2) no bend at the waist. Those two levels were collapsed into one criterion in Version 2: “slight or no bend at the waist”.

Half of panelists reported needing modifications for Criterion A to define the degree of knee bend, 3 (38%) reported needing modifications for Criterion G to define the degree of knee bend, and 5 (63%) reported needing modifications for Criterion H to define degree of bend at waist and clarify language. In addition, there were scoring discrepancies for all 3 criteria during the rating exercises, with some panelists selecting one level of severity and others selecting the other for the same participant. Since the difference between the two severity levels for each of the three criteria were difficult to define and observe, they were collapsed into one level to improve scoring reliability and discussed during Round 2.

### Modified Delphi process: Round 2

During the in-person discussion, the scorecards for all movement tasks, except Run, were discussed. Due to time limitations, the meeting ended before the panel discussion of Run, but a subset of the Delphi panel (n = 3) with availability met after the in-person discussion to discuss the Run task. For all scorecards, 100% of the panelists agreed on all changes made to the scorecards during Round 2. A universal change was made to the structure of all scorecards to add a level 0 sub-criterion to all compensatory movement criteria so that physical therapists could indicate when a compensation was not present.

There were 47 changes (31%) made to the wording of 150 compensatory movement sub-criteria to improve understandability. As an example of the types of wording changes made during Round 2, the Stand Up from Sitting on Floor (**Table 3**) criterion B (“walking hands along floor”) from Version 2 was changed to “walking hands towards the body along the floor before lifting torso”. The words “towards the body” and “before lifting torso” were added to clarify the exact movement compensation that raters should identify and avoid confusion about how and when the hands walk along the floor.

Twenty compensatory movement sub-criteria were added. The panelists identified when compensatory movements were missing from the scorecards. As an example of an added sub-criterion, a compensation in the Climb 5 Stairs scorecard involved a spectrum of severity for body positioning on the stairs that was missing a higher level of severity that panelists had observed in practice. The highest level of severity in Version 2 was “turning torso to face wall or railing to side step”, and the panelists voted unanimously to add the compensation “uses rail to bear weight” as a missing higher level of severity that occurs as muscles weaken.

Thirty compensatory movement sub-criteria were removed. When evaluating each compensatory movement on the scorecards, panelists were asked to ensure that each movement included was a clinically meaningful compensation. Of the 30 sub-criteria that were removed, 13 were removed to ensure the clinical meaningfulness of each movement (detailed in **S1 Table** and representative example provided in **Table 4**), 6 were replaced with related compensations, 3 were integrated into other criteria, and 8 were removed from Shift Weight in Bed and Arms Off and On Armrests since the part of the task they applied to was removed. For the Shift Weight in Bed task, the participant was originally instructed to sit up in bed, lean forward, and then lean to each side. During Round 2, the panelists unanimously decided that leaning to each side is not as functionally important to the task and that it should be removed, which resulted in the removal of the 5 criteria associated with leaning to each side. For the Arms Off and On Armrests task, the scorecard originally included evaluation of both taking the arm off the armrest and putting it back on. During Round 2, the panelists unanimously recommended removal of the 3 criteria associated with the gravity-assisted motion of lowering the arm off the armrest.

**Table 4:** Representative example of removal of compensatory movement criteria to ensure clinical meaningfulness during Round 2.

Movement Task	Change to Scorecard to Improve Clinical Meaningfulness of Criteria	Representative Quotes from Panelists
Walk	Removed “loss of functional arm swing”	“I thought with some of the arm swing stuff, what does it mean? I feel like the driving factor is somewhere else that we’ve talked about.”
		“Since it’s not driving a compensation, it’s more variable. You see it all the time ‘go ahead and walk’ and because the kids are in a testing environment, they’re rigid and they’re not really moving their arms and it has nothing to do with a compensation. It’s just that you’ve put them in a more structured environment…I think you’re going to have a lot of variability in what arm swing they do because there is not a compensation driving it.”

## Discussion

This study developed and refined, using a modified Delphi approach, the DVA scorecards. The modified Delphi process was critically important to the revision of the scorecards to ensure that the compensatory movement criteria are understandable, clinically meaningful, and comprehensive. Understandability of the compensatory movement wording is essential for high inter-rater and intra-rater scoring reliability, which was tested in another study [20]. By delineating between different severity levels of compensated movement, the DVA provides an opportunity to detect incremental functional changes that occur in a shorter duration of time. Those incremental functional changes are only valuable to measure if they are clinically meaningful.

The DVA scorecard development utilized physical therapists, with expertise in the evaluation of movement patterns used by patients with DMD, as reporters of clinical meaningfulness and comprehensiveness of the selected compensatory movement criteria. Patients may not be aware of their compensatory movements since the movements develop in response to progressive muscle weakness and contracture [26, 27], and caregivers may not be able to articulate the specific compensatory movement patterns. Physical therapists understand the functional importance of each movement compensation as it relates to muscle weakness and contracture, and they have observed these patterns adopted by the breadth of patients they have evaluated.

The DVA is intended to assess clinical benefit in clinical trials by evaluating the impact of a potential therapeutic on participants’ daily function in the home environment. Existing functional assessments for DMD measure performance in a clinical setting, which may not be reflective of a patient’s daily ability as it can be influenced by patient effort in clinic, encouragement by medical staff or caregivers, and travel-related fatigue [2]. The DVA includes functional tasks that those with DMD perform in their daily lives and measures their typical performance in their home environment. The COVID-19 pandemic called attention to the need for clinical outcome assessments that can be administered remotely [28, 29] and accelerated a trend towards remote clinical trials [30]. Existing functional measures require frequent travel to clinical sites for evaluation by physical therapists, while the DVA mobile application allows caregivers to collect patients’ functional data securely in the home environment. Participation in clinical trials can pose physical, emotional, and logistical challenges for people with DMD and their families [31], and use of the DVA at home removes the travel and financial burden placed on patients and families.

This study was not without limitations. First, only a subset of panelists discussed the Run movement task during Round 2 due to time limitations during the in-person discussion. While it is possible that the panelists who were not present during the Run discussion may have had differing views, the subset of panelists who were available agreed on the changes that were made to the scorecard. Second, this study only included panelists who work in the United States, and it is possible that the understandability of the compensatory movement descriptions may be different in other countries; however, since the DVA is scored using central raters, it is possible for physical therapists who work in the United States to score videos collected from other countries.

## Conclusions

Physical therapists with extensive experience evaluating patients with DMD confirmed that the compensatory movement criteria included in the DVA scorecards were understandable, comprehensive, and clinically meaningful. The use of the DVA in clinical trials has the potential to expand patient access to studies by eliminating the need to travel to clinical sites and including movement tasks that can be assigned to participants at any stage of their disease. Future research will evaluate whether the DVA is able to detect functional changes in a shorter duration than existing measures, which could reduce the time required of patients for clinical trial participation.

## Supporting information

S1 Table All instances of removal of compensatory movement criteria to ensure clinical meaningfulness during Round 2. (DOCX)
