# Supplementary material for: Development of Duchenne Video Assessment scorecards to evaluate ease of movement among those with Duchenne muscular dystrophy
Source: PLoS One. 2022 Apr 13;17(4):e0266845. doi: 10.1371/journal.pone.0266845 (PMC9007341; doi:10.1371/journal.pone.0266845)
Supplement: S1 Table — (DOCX) [file pone.0266845.s001.docx]

| **Movement Task** | **Change to Scorecard to Improve Clinical Meaningfulness of Criteria** | **Representative Quotes from Panelists** |
| --- | --- | --- |
| Climb 5 Stairs | Removed “leaning torso toward wall or railing while facing upwards” | “There’s a lot more room for variability with the three [levels].”  “It doesn’t look like a clear progression. It seems a little bit like a strategy.” |
| Walk | Collapsed 2 severity levels for base of support into 1 level | “Pudgy healthy boys need to be wider [base of support]…I think ‘wider than shoulder-width’.”  “Because that’s when it starts making a difference too, when you’re starting out here not the difference between this [demonstrating wider than shoulder-width and shoulder-width].” |
|  | Collapsed 2 severity levels for lordosis into 1 level | “It gets tricky when you’re using mild, moderate, severe.”  “Unless it’s a comparative thing.”  “I think it’s an important criterion. We get it, but how do you quantify it?”  “The question is what is moderate and what is severe?” |
|  | Collapsed shoulder sway and head/neck sway into “waddle with trunk sway” | “I think what you’re really trying to get from the shoulders is the trunk. And as PTs, I think we describe trunk sway more than shoulder sway. When the trunk sways, the shoulders move. But you can have some other movement of the shoulders that has nothing to do with the trunk. And I think it’s the trunk sway you’re trying to pick up on.”  “My guess is that not everyone does the same amount of head sway for the amount of trunk sway they are doing. Those short necks won’t sway as much as those long necks.”  “As PTs, we don’t call it head [sway] because we see it in the trunk.”  “The head just kind of follows unless it is the driving momentum.”  “The waddle is the way it starts, and the sway is the way it ends.”  “You don’t have a waddle without a sway.” |
|  | Removed “loss of functional arm swing” | “I thought with some of the arm swing stuff, what does it mean? I feel like the driving factor is somewhere else that we’ve talked about.”  “Since it’s not driving a compensation, it’s more variable. You see it all the time ‘go ahead and walk’ and because the kids are in a testing environment, they’re rigid and they’re not really moving their arms and it has nothing to do with a compensation. It’s just that you’ve put them in a more structured environment…I think you’re going to have a lot of variability in what arm swing they do because there is not a compensation driving it.” |
| Run | Removed “external hip rotation” | No quotes available (discussion occurred outside of recorded Round 2 meeting) |
|  | Removed “greater than typical lumbar lordosis” |  |
|  | Removed “Limited heel lift on trailing leg as it starts to swing through” |  |
| Jump Forward | Removed hip flexion during the take-off and landing phases (3 sub-criteria) | “You can’t bend your hips if you can’t bend your knees. I think those two are redundant…they are too related.”  “If you bend your knee, you are going to have to bend your hips. And to stay upright, your trunk comes forward.” |
|  | Collapsed 2 severity levels for arm swing into one level | “I think [criteria] C and E are really closely linked because you’re not going to be able to bring your arms through unless you hit C…do you want to combine them into one?...It is so dependent on his start position. He never brings them back to swing them through.”  “I agree that I think it is related to the start. Because you can’t really bring them forward if you don’t bring them back…I think those two are related” |
| Raise Hands Above Head | Collapsed 2 severity levels for elbow flexion into one level | “A little bit of bend versus a lot, how much do you think that affects their ability?”  “If you have a kid, you’re having him do the PUL, right? And they come up and over, and they bend their arms. Do I see a big functional change between this amount of bend or this amount of bend? So is the question whether they’re just bending their arms to shorten the lever arm or is the question are they bending their arms greater than 90 or less than 90?...I’m just wondering if it’s just shortening the lever arm, bending the elbows.”  “If they shorten their arms but ultimately get all the way up there, does it [degree of flexion] matter as much? Or should we just say do they have to shorten their arc to get up here?” |
